# Neural Androgen Receptor Deletion Impairs the Temporal Processing of Objects and Hippocampal CA1-Dependent Mechanisms

**DOI:** 10.1371/journal.pone.0148328

**Published:** 2016-02-05

**Authors:** Marie Picot, Jean-Marie Billard, Carlos Dombret, Christelle Albac, Nida Karameh, Stéphanie Daumas, Hélène Hardin-Pouzet, Sakina Mhaouty-Kodja

**Affiliations:** 1 Neuroscience Paris Seine, Institut National de la Santé et de la Recherche Médicale, Unité Mixte de Recherche (UMR) S1130, Université P. et M. Curie, Paris, France; 2 Centre National de la Recherche Scientifique, UMR 8246, Université P. et M. Curie, Paris, France; 3 Sorbonne Universités, Université P. et M. Curie UM CR18, Université Paris 06, France; 4 Centre de Psychiatrie et Neurosciences, Université Paris Descartes, Sorbonne Paris Cité, UMR 894, Paris, 75014 France; Centre national de la recherche scientifique, University of Bordeaux, FRANCE

## Abstract

We studied the role of testosterone, mediated by the androgen receptor (AR), in modulating temporal order memory for visual objects. For this purpose, we used male mice lacking *AR* specifically in the nervous system. Control and mutant males were gonadectomized at adulthood and supplemented with equivalent amounts of testosterone in order to normalize their hormonal levels. We found that neural *AR* deletion selectively impaired the processing of temporal information for visual objects, without affecting classical object recognition or anxiety-like behavior and circulating corticosterone levels, which remained similar to those in control males. Thus, mutant males were unable to discriminate between the most recently seen object and previously seen objects, whereas their control littermates showed more interest in exploring previously seen objects. Because the hippocampal CA1 area has been associated with temporal memory for visual objects, we investigated whether neural *AR* deletion altered the functionality of this region. Electrophysiological analysis showed that neural *AR* deletion affected basal glutamate synaptic transmission and decreased the magnitude of N-methyl-D-aspartate receptor (NMDAR) activation and high-frequency stimulation-induced long-term potentiation. The impairment of NMDAR function was not due to changes in protein levels of receptor. These results provide the first evidence for the modulation of temporal processing of information for visual objects by androgens, via AR activation, possibly through regulation of NMDAR signaling in the CA1 area in male mice.

## Introduction

Several evidences strongly suggest that testosterone plays a neuromodulatory role in cognitive functions [1,2]. In humans, hypogonadism affecting young adults, chemically castrated individuals or older men [3–6] is associated with spatial, visual, verbal and episodic memory defects [4,7–9]. In rodents, performances in spatial learning and memory abilities such as object recognition, fear conditioning and spatial memory tasks are decreased by castration and restored by testosterone replacement [10–13]. In this context, no studies addressed a potential modulation by testosterone of temporal processing of information. Temporal processing of information is the ability to remember the order in which items or events have been experienced. This component of episodic memory is impaired in neurodegenerative diseases, such as Alzheimer’s disease [14]. Patients with relatively selective hippocampal damage have impaired temporal order memory for visual objects, linguistic information and spatial locations [15,16]. The perirhinal and prefrontal cortices and the hippocampus are required for the processing of temporal order information [17–20]. The components of this neural system function in a cooperative way to retrieve the information required for long-term temporal order memory, whereas the hippocampus alone plays a major role in short and intermediate-term memory processes [17,18,21]. In particular, the integrity of the Cornu Ammonis (CA) 1 area is required for the expression of temporal memory for sequential non-spatial events, such as visual objects [22,23].

In the male nervous system, testosterone can act directly or through its non-aromatizable metabolite, dihydrotestosterone, to activate the androgen receptor (AR). Testosterone can also be locally aromatized into estradiol, which then stimulates estrogen receptors. Estrogen-mediated regulation of cognitive behavior and synaptic plasticity has been largely reported in male rats. Intra hippocampal injection of estradiol enhanced memory in a spatial water maze task, possibly through an interaction with muscarinic cholinergic systems [24]. Acute treatments using estrogens, estrogen receptor agonist or selective estrogen receptor modulators were shown to facilitate long-term potentiation (LTP) in adult hippocampal slices [25], affect the number and shape of dendritic spines in CA1 pyramidal neurons [26] and decrease thorn density of hippocampal CA3 neurons [27]. Estradiol through both estrogen receptors α and ß (ERα and ERß) was also reported to regulate N-methyl-D-aspartate receptor- (NMDAR) mediated transmission and thus synaptic plasticity in the dentate gyrus of juvenile males [28]. In mice, ERß activation improved performance in hippocampus-dependent memory tasks, enhanced long-term potentiation in hippocampal slices of wild-type but not *ERß* knockout mice and increased dendritic branching and density of mushroom-type spines [29]. *ERß* knockout males also exhibited memory impairment in a hippocampus-mediated fear-conditioning paradigm [30]. Finally, activation of hippocampal ERα after learning impaired memory formation in contextual fear conditioning tasks [31].

AR function has been investigated less thoroughly, although this receptor is known to be expressed in the cortex and hippocampus with a predominant distribution in CA regions [32,33]. Studies based on gonadectomy and dihydrotestosterone supplementation or the use of androgen-insensitive rats carrying the testicular feminization mutation (*Tfm*) have suggested that the AR mediates the androgen-induced maintenance of normal spine synapse density in the CA1 area and prefrontal cortex [34,35]. It modulates the functional properties of pyramidal cells in both the CA1 [36] and CA3 areas [37] and increases CA3 dendritic thorns in hippocampal slices [38].

In this study, we investigated the role of androgens through the neural AR in temporal order memory for visual objects and in functional properties of the underlying hippocampal CA1 area. For this purpose, we used a mouse model lacking *AR* selectively in the nervous system [33] in order to analyze the effects of specific neural *AR* mutation on temporal order memory for visual objects, novel object recognition, anxiety-related behaviors and circulating levels of corticosterone. Electrophysiological studies were conducted to test the functionality of the CA1 area, where the protein amounts of glutamate receptors were also quantified.

## Materials and Methods

### Animals

Three months-old males floxed for the *AR* and carrying (mutants, AR^NesCre^) or not (controls, AR^fl^/Y) the Cre recombinase under the control of promoter and nervous system-specific enhancer of nestin (Nes) were obtained in a C57BL/6J genetic background as previously described [33,39]. After weaning, control and mutant males obtained in the same litters were group-housed under a 12:12-h light–dark cycle and maintained at 22°C. They were fed with a standard diet with free access to food and water. The presence of the Cre transgene was determined by polymerase chain reaction analysis as previously described [33]. This work was performed in accordance with the French and European legal requirements (Decree 2010/63/UE) and was approved by the “Comité d'éthique en expérimentation animale” Charles Darwin N°5 (project number 01490–01). Euthanasia was carried by intra-peritoneal injection of pentobarbital (150–200 mg/kg).

### Gonadectomy and treatment

Due to the altered negative feedback exerted by testosterone, mutant males exhibit higher hormonal levels compared to their control littermates [33]. Therefore, in order to normalize testosterone levels between control and mutant males, all males used in this study were subjected to gonadectomy under general anesthesia (xylazine 10 mg/kg / ketamine 100 mg/kg) and supplemented with subcutaneous Silastic® tubes containing 10 mg of testosterone (Sigma–Aldrich). Males were subcutaneously injected with the analgesic buprenorphine (0.05 mg/kg) after surgery. Measurement of circulating levels of testosterone two-four weeks later showed similar amounts between control and mutant males (8.8 ± 0.31 ng/ml and 9 ± 1.8 ng/ml, respectively) as previously described [39]. Experiments were conducted between two and four weeks after gonadectomy and treatment.

### Effects of AR^NesCre^ mutation on hippocampal AR and ERα amounts

**Fig 1:**
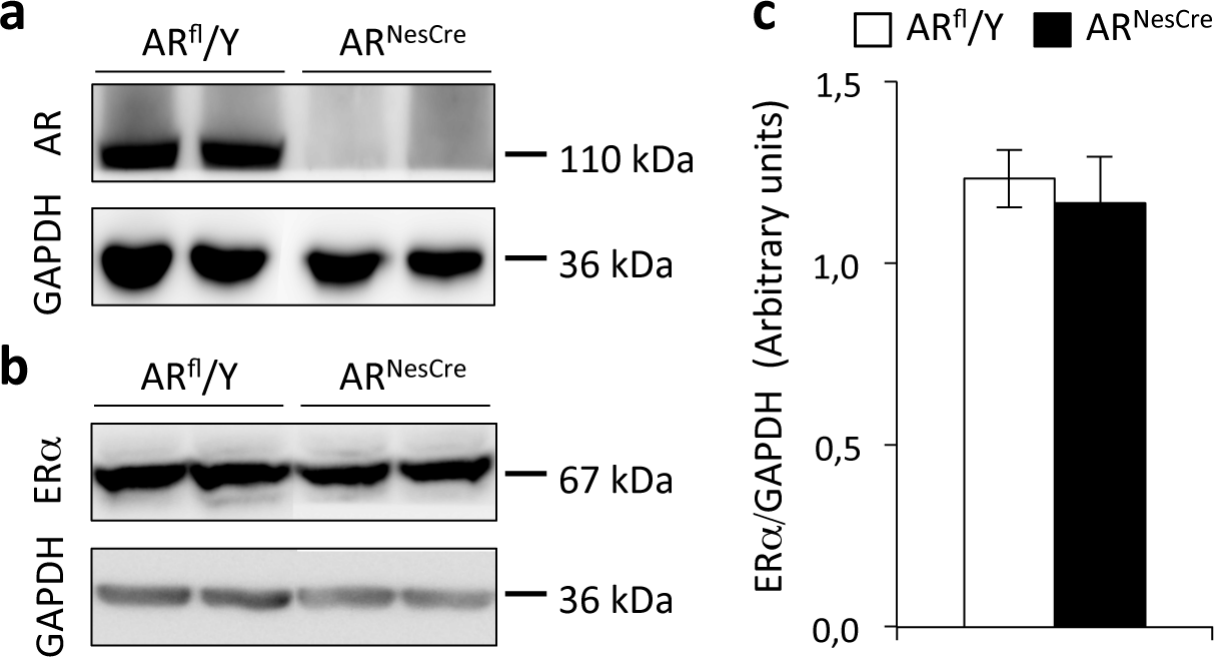
Western blotting of AR and ERα in the hippocampus of AR^fl^/Y and AR^NesCre^ male mice. (**a-b**) Immunoblotting of hippocampal AR (**a**), ERα (**b**) and GAPDH proteins (**a**, **b**) in control (AR^fl^/Y) and mutant (AR^NesCre^) males. (**c**) Quantification of hippocampal ERα protein normalized to GAPDH in 6 males per genotype.

Proteins (30 μg) were extracted from the hippocampus, separated on a 7.5% polyacrylamide gel and transferred onto nitrocellulose membranes as previously described [33]. The blots were probed overnight at 4°C with polyclonal rabbit (1/400 anti-AR #sc-816 and 1/400 anti-ERα #sc-542; Santa-Cruz Biotechnology) or monoclonal mouse antisera (1/10000 anti-glyceraldehyde 3-phosphate dehydrogenase gene (GAPDH) #sc-32233 from Santa-Cruz Biotechnology). They were then incubated with horseradish peroxidase-conjugated secondary anti-rabbit #111.035.144 or anti-mouse #115.035.062 antisera (1/5000, Jackson Immunoresearch). Signals were visualized with pico- (for GAPDH) and femto- (for AR and ERα) Super Signal detection kit (Thermo Scientific), quantified with ImageJ software (NIH) and normalized with respect to GAPDH. As we have previously shown by immunohistochemistry [33], AR protein was detected in the hippocampus of control (AR^fl^/Y) males but not in their mutant (AR^NesCre^) littermates (Fig 1A). By contrast, hippocampal ERα protein was present in both AR^fl^/Y and AR^NesCre^ males (Fig 1B). Quantification of ERα protein normalized to GAPDH showed similar amounts in the two genotypes (Fig 1C).

### Behavioral tests

Tests were conducted during the second half of the light phase and videotaped for further analyses. All behaviors were scored blind to genotype.

#### Object recognition tests

**Fig 2:**
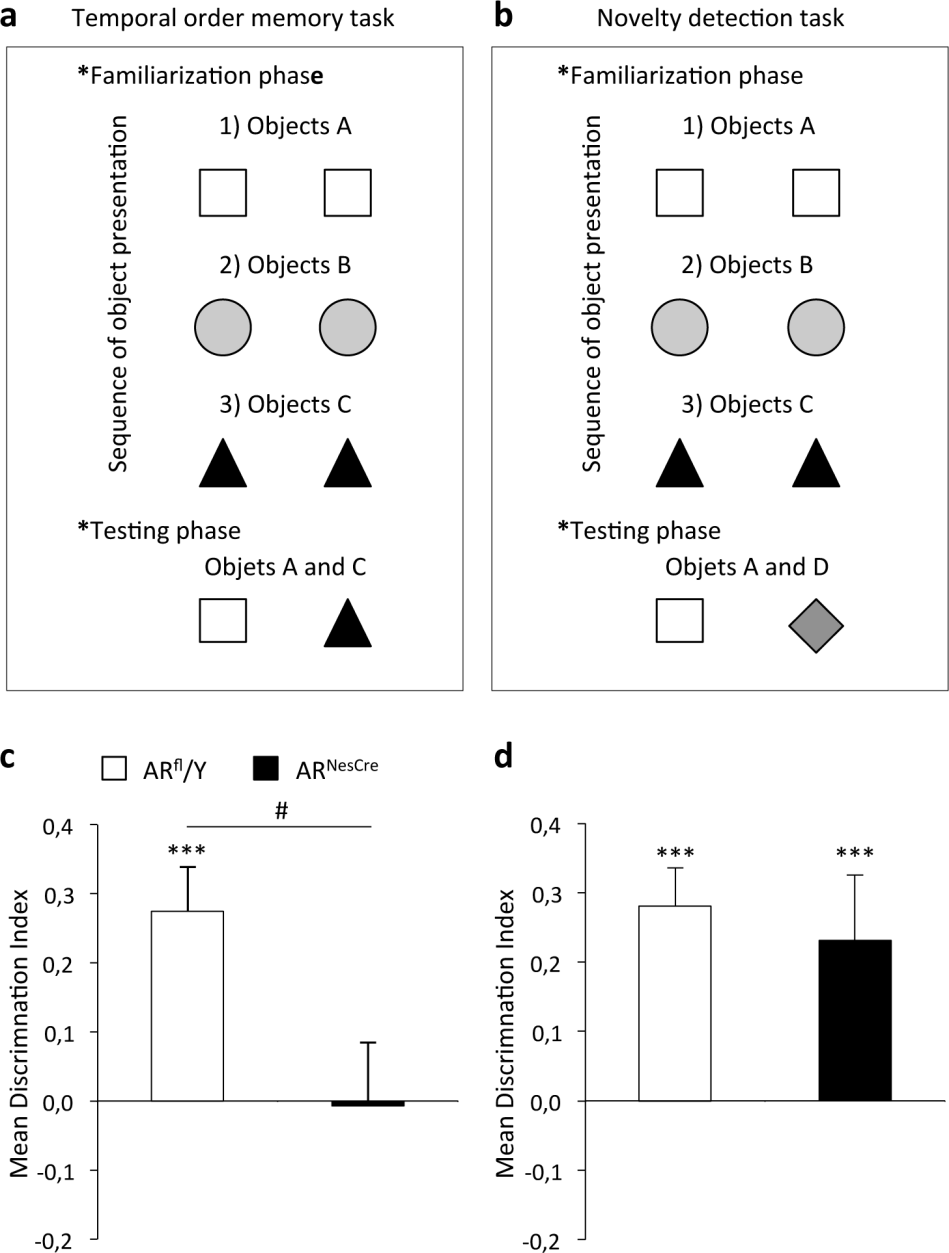
Temporal order memory and object recognition in AR^fl^/Y and AR^NesCre^ male mice. (**a-b**) Familiarization and testing phases of the temporal order (**a**) and novelty detection (**b**) tasks. (**c-d**) Discrimination Indexes in the temporal order (**c**) and novelty detection tasks (**d**) for males (n = 8–9 males per genotype). ***p < 0.001 *versus* object A; ^#^*p* < 0.05 *versus* controls.

The object recognition task was conducted in a round green plastic arena (45 cm diameter) lined with sawdust. The task had three phases: habituation, familiarization and testing. The habituation phase consisted of two days during which the animals were exposed to the arena in the absence of objects (a first 30 min session and two daily 10 min sessions 3 h apart) and one day of habituation to objects in place in the arena to decrease neophobia. The familiarization phase consisted of three sessions during which mice were allowed to explore two objects (A–A, B–B, and C–C) for at least 15 seconds per object (Fig 2A). An interval of 3 minutes was left between sessions. Ten minutes after the last session (objects C), the males were returned to the arena for a five-minute testing phase, in which they were placed in the presence of a copy of one object each from the first (A) and last (C) sessions. In the novelty detection task, the habituation and familiarization phases were as described above. By contrast, during the testing phase, mice were exposed to a familiar object (A) and a new object that they had not seen before (D) (Fig 2B). Exploration was defined as the time spent exploring the object. We assessed the discrimination index (DI) between objects previously defined [40] as the absolute difference in the time spent exploring the novel (*T*_N_) and the familiar objects (*T*_F_) divided by the total time spent exploring the objects [DI = (*T*_N_ − *T*_F_)/(*T*_N_ + *T*_F_)].

#### Analysis of anxiety-related behavior

The elevated plus maze apparatus consisted of a gray plastic maze composed of two opposite open arms (30 cm length, 7 cm width, 67 cm height) and two opposite closed arms of the same size enclosed by 17 cm-high walls. The elevated zero maze apparatus consisted of a circular maze (56 cm diameter, 7 cm width, 67 cm height) divided in two closed arms interconnected by two open arms (44 cm length). Males were placed in the center of the elevated plus maze maze, between the two closed arms, facing an open arm, or in the closed arms for the elevated zero maze and were allowed to explore the maze freely for nine minutes. The latency to enter and the time spent in the open arms were analyzed. A mouse was considered in the open arm when the 4 paws were introduced. The intensity of light was 30 lux.

### Corticosterone levels

One week after the end of behavioral tests, basal corticosterone levels were monitored by collecting blood from the tail vein into heparinized capillary tubes at 8–9 am and 6–7 pm. To avoid the effect of stress, the blood collection procedure was limited to 1 min. Blood was centrifuged at 2000 rpm for 15 min and the resulting plasma was frozen until the assay. Corticosterone levels were determined with a radioimmunoassay kit (MP Biomedicals).

### Electrophysiology

A second group of mice was used to perform electrophysiological studies. Transverse hippocampal slices (400 μm) were used [41]. Basal synaptic transmission, supported by non-NMDAR activation, was investigated by extracellular recordings for the apical dendritic layer of the CA1 area, with micropipettes filled with 2 M NaCl. Presynaptic fiber volleys and field excitatory postsynaptic potentials were evoked at 10-second intervals, by electrical stimulation of the Schaffer collaterals. The averaged slope of three successive responses was determined with Win LTP software [42] and plotted against stimulus intensity (300 to 500 μA), to obtain input/output curves. The specific activation of NMDAR was assessed by recording potentials in slices perfused with low-Mg^2+^ (0.1 mM) medium supplemented with the non-NMDAR antagonist 2,3-dioxo-6-nitro-1,2,3,4-tetrahydrobenzoquinoxaline-7-sulfonamide (10 μM). The paired-pulse facilitation of synaptic transmission was induced by the electrical stimulation of afferent fibers with paired pulses (inter-stimulus interval: 30 ms). Paired-pulse facilitation was assessed by dividing the slope of the second response by that of the first response.

Long-term potentiation (LTP) of synaptic transmission was investigated in the hippocampal CA1 area following different paradigms of conditioning stimulation. First, theta-burst conditioning stimulation was induced after 15 minutes of baseline recording, consisting of trains of four pulses at 100 Hz, separated by 200 ms, this sequence being repeated four times, with an interval of 10 s between sequences. We also evaluated the effects of high-frequency stimulation consisting of two high frequency trains (100 Hz for 1 s) separated by a 20 s interval. Long-term depression (LTD) was assessed by low-frequency conditioning stimulation, at 2 Hz, for 10 min. In both LTP and LTD investigations, testing with a single test pulse was resumed for 60 min after the conditioning stimulus, to determine the level of synaptic plasticity.

### Quantification of glutamate receptors in the CA1 region

Hippocampi were dissected to separate the CA1 area. Proteins (10–20 μg) were then extracted from the CA1 area, separated on a 7.5% polyacrylamide gel and transferred onto nitrocellulose membranes as previously described [43]. The blots were probed overnight at 4°C with polyclonal rabbit (1/1000 anti-GluN2A #07–632 and 1/1000 anti-GluR2 #AB1768; Millipore) or monoclonal mouse antisera (1/200 anti-GluR1 #04–855, 1/1000 anti-GluN2B #05–920 and 1/1000 anti-GluN1 #05–432 from Millipore; 1/5000 anti-ß-actin #A5441 from Sigma-Aldrich). They were then incubated with horseradish peroxidase-conjugated secondary anti-rabbit #111.035.144 or anti-mouse #115.035.062 antisera (1/5000, Jackson Immunoresearch). Signals were visualized with enhanced chemiluminescence detection kit for ß-actin (GE Healthcare) and Super Signal detection kit (Thermo Scientific) for the other proteins, quantified with ImageJ software (NIH) and normalized with respect to ß-actin.

### Statistics

Values are presented as means ± S.E.M. Data were analyzed by one-way analysis of variance (ANOVA) for the effect of genotype, separate univariate t tests with 0 as the reference value for object recognition, and repeated-measures ANOVA followed by Tukey’s post hoc tests for synaptic plasticity. The significance threshold was fixed at 5%.

## Results

### Temporal order memory and novelty detection tasks

The total time spent exploring the two objects did not differ significantly between the genotypes (16 ± 2 s *versus* 24 ± 4 s in the temporal memory task, and 26 ± 3 s *versus* 27 ± 3 s in the novelty task, for AR^fl^/Y and AR^NesCre^ males, respectively). However, in the temporal order task for visual objects, AR^fl^/Y mice were more interested (*p* = 1.62.10^−5^) in exploring object A than object C (Fig 2C). This is consistent with previous findings showing that rats select the object that was presented earlier when faced with two objects in a similar paradigm of recency discrimination [18,22,44]. By contrast, AR^NesCre^ mice did not discriminate between the objects presented. ANOVA revealed a significant main effect of genotype (F_(1,15)_ = 6.55, *p* = 0.021).

In the novelty detection task, analysis of mean scores showed that both AR^fl^/Y and AR^NesCre^ males spent more time exploring the new object than the familiar object (*p* = 1.8.10^−6^ and *p* = 0.003, respectively) as shown in Fig 2D. A one-way ANOVA revealed no effect of genotype (F_(1,15)_ = 0.22, *p* = 0.65).

### Anxiety-related behavior and corticosterone levels

**Fig 3:**
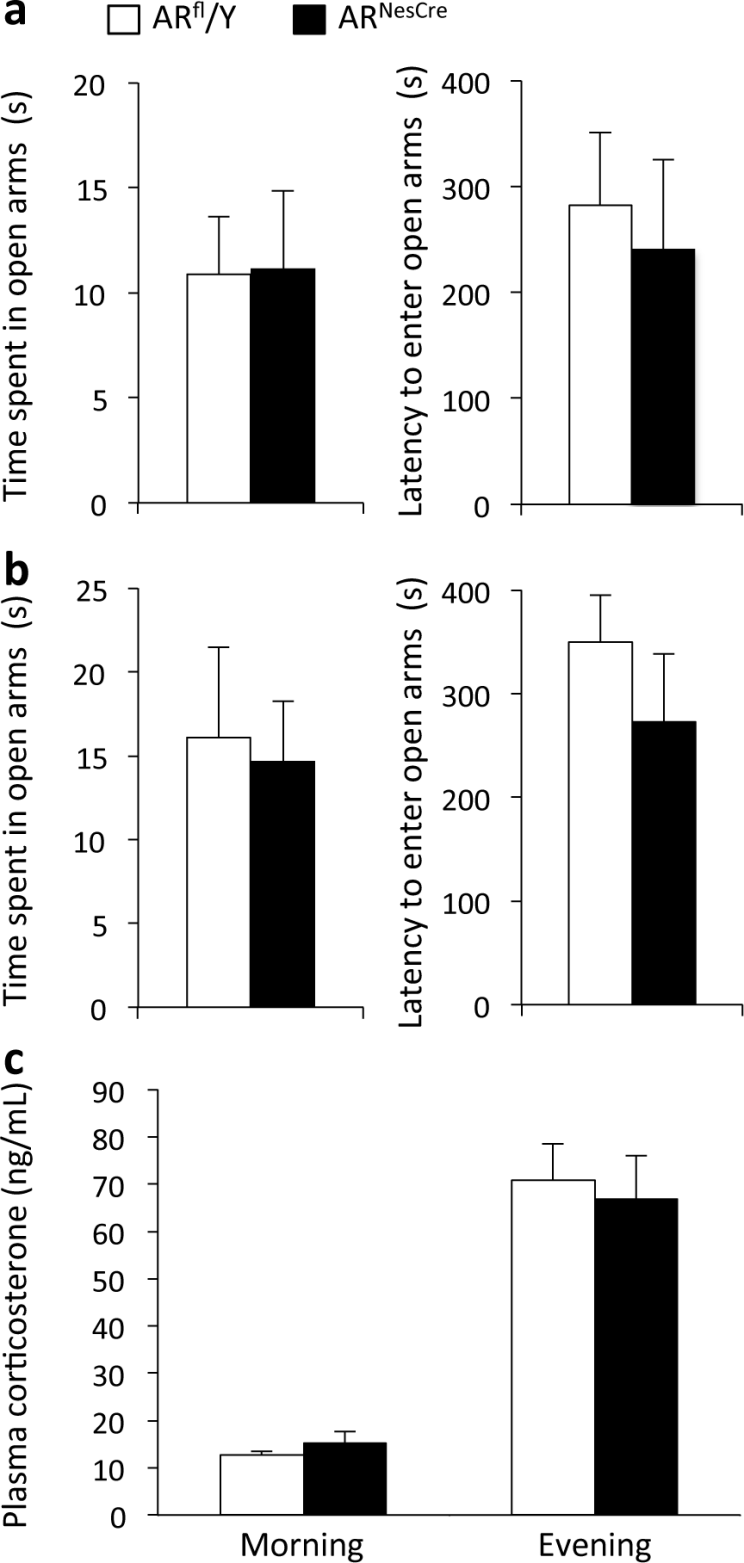
Anxiety state level and corticosterone levels in AR^fl^/Y and AR^NesCre^ male mice. **(a-b)** Time spent and latency to enter in the open arms of the EPM (**a**) or the O-maze (**b**) for controls and mutants (n = 8–11 males per genotype). (**f**) Corticosterone secretion during the circadian cycle (n = 8–11 males per genotype).

We explored whether AR^NesCre^ mutation altered anxiety-related behavior or regulation of the hypothalamic-pituitary-adrenal axis, thereby potentially affecting cognition. In the elevated plus maze, the latency to enter (F_(1,15)_ = 0.281, *p* = 0.60) and the percentage of time spent (F_(1,15)_ = 0.03, *p* = 0.96) in the open arms of the maze were similar for the two genotypes (Fig 3A). In the elevated zero maze test, the latency to enter (F = 1.004, *p* > 0.05) and the percentage of time spent (F = 0.043, *p* > 0.05) in the open arms of the maze were also comparable between AR^fl^/Y and AR^NesCre^ males (Fig 3B).

Basal corticosterone secretion followed a circadian rhythm, with low levels in the morning and higher levels in the evening (Fig 3C), and no effect of genotype (F_(1,17)_ = 0.016; *p* = 0.90).

Together, these findings indicate that AR^NesCre^ mutation selectively impairs the processing of temporal information for visual objects, without affecting the detection of new visual objects or anxiety-like behavior. As the CA1 area is involved in temporal memory for visual objects [22,23], we investigated whether AR^NesCre^ mutation altered the functioning of this particular region.

### Electrophysiological studies

#### Basal neurotransmission

**Fig 4:**
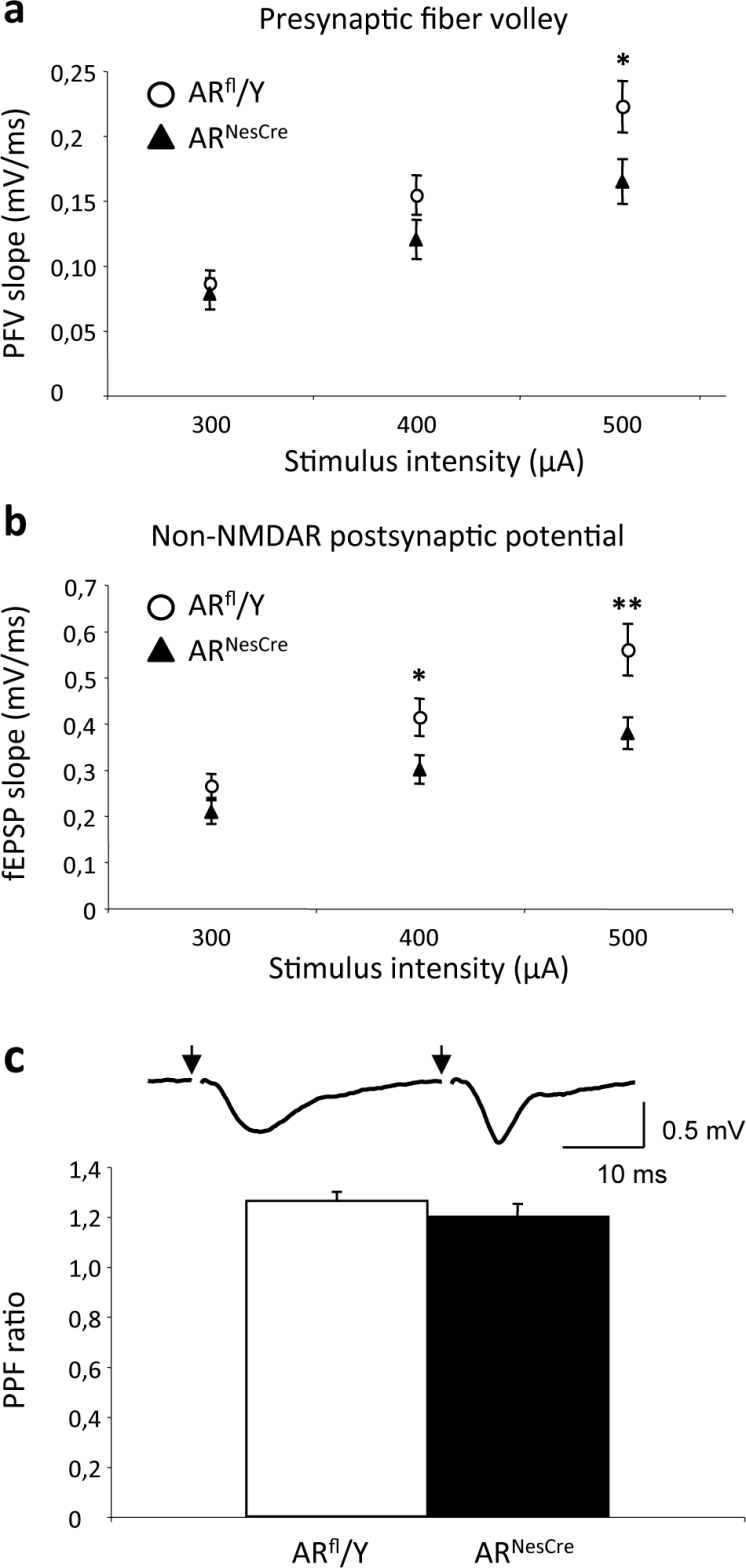
Basal synaptic transmission in the hippocampal CA1 area of AR^fl^/Y and AR^NesCre^ male mice. (**a-b**) Input/output (I/O) curves of presynaptic fiber volleys (PFVs) (**a**) and field excitatory postsynaptic potentials (fEPSPs) (**b**) induced in control medium by the electrical stimulation of glutamatergic afferents (44 slices from 8 males per genotype). PFV magnitude was significantly smaller in AR^NesCre^ males only at the highest stimulus intensity (*p < 0.05), whereas fEPSP values were already lower at lower intensities (**p < 0.01). (**c**) Upper panel, representative paired-pulse facilitation (PPF) of fEPSPs induced in a AR^NesCre^ male by two successive stimuli (arrows) delivered with a 30 ms inter-stimulus interval. Lower panel, mean PPF magnitude (28–29 slices per genotype).

A comparison of input/output curves showed that presynaptic fiber volleys were significantly lower in AR^NesCre^ males, but only for the 500 μA stimulus, whereas field excitatory postsynaptic potentials were already significantly reduced at a lower stimulus intensity (Fig 4A and 4B). AR^NesCre^ mutation therefore affects basal glutamate synaptic transmission in the CA1 area through changes in the density and/or functional properties of postsynaptic non-NMDAR on pyramidal cells, but also through modifications in the presynaptic glutamate afferents when a strong activation was applied. By contrast, paired-pulse facilitation was not affected in AR^NesCre^ animals (Fig 4C) indicating that neural *AR* deletion does not alter the mechanisms underlying glutamate release.

#### Synaptic plasticity

**Fig 5:**
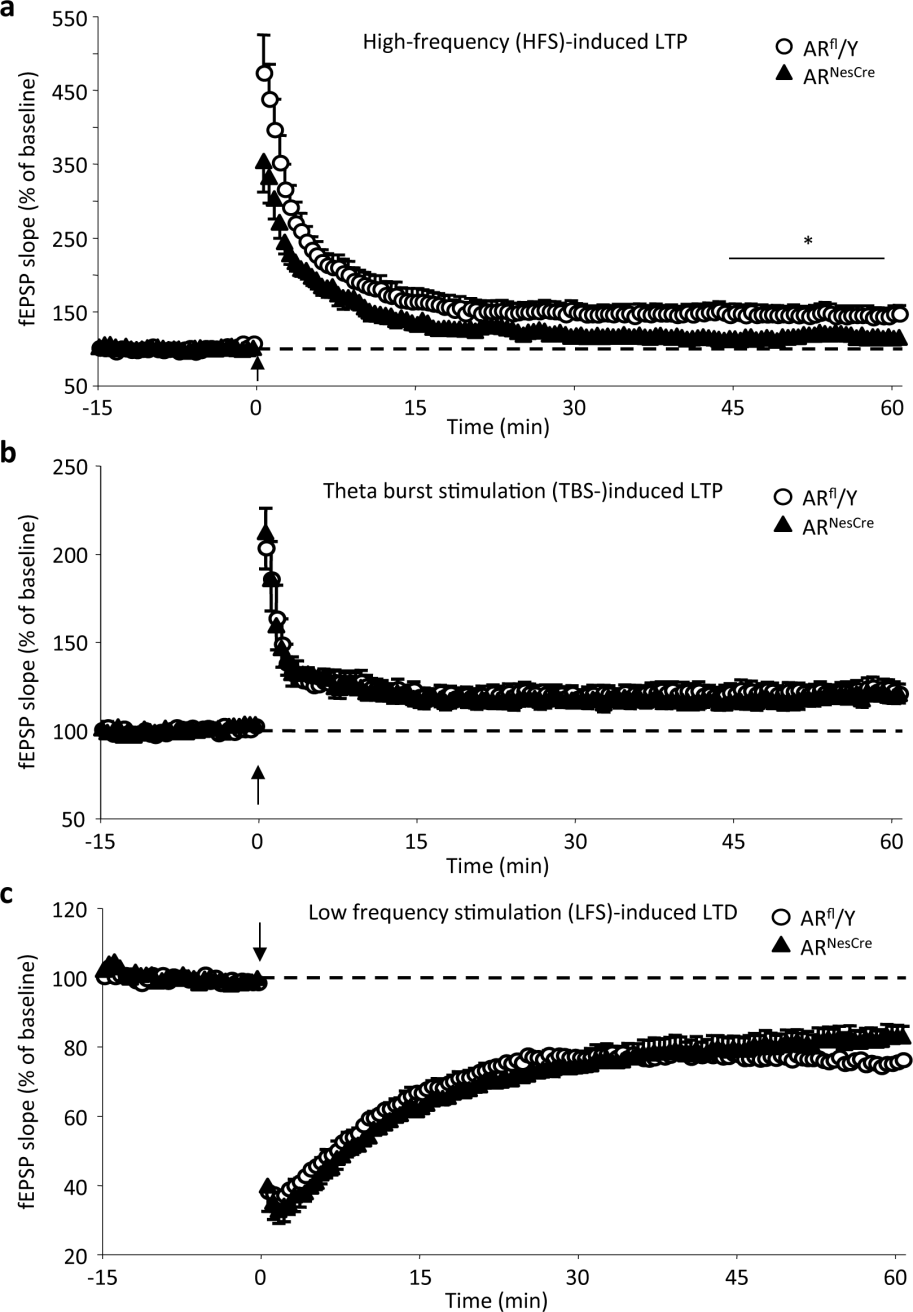
Synaptic plasticity in the hippocampal CA1 area of AR^fl^/Y and AR^NesCre^ male mice. (**a-b**) Comparison of the time course of mean long-term potentiation (LTP) induced by high-frequency stimulation (HFS) (**a**; 9 slices from 7 animals per genotype) or by theta-burst stimulation (TBS) (**b**; 12 slices from 7–8 males per genotype). *p < 0.05 *versus* control. (**c**) Comparison of the time course of mean long-term depression (LTD) induced by low-frequency stimulation (LFS) in males (10–11 slices from 5–6 animals per genotype).

In slices from both AR^fl^/Y and AR^NesCre^ males, high-frequency stimulation induced a significant increase in field excitatory postsynaptic potentials slope (Fig 5A), which persisted until the end of recording (stimulus effect, *p* = 0.001 and 0.03 for AR^fl^/Y and AR^NesCre^ mice, respectively). However, the mean LTP magnitude determined for the last 15 minutes of recording, between 45 and 60 minutes after the conditioning stimulation, was significantly higher (*p* = 0.04) in control (45.3 ± 11.6%) than in mutant (16.2 ± 7.1%) males. No difference was found in the magnitude of synaptic plasticity between groups of mice when theta-burst conditioning stimulation-induced LTP (Fig 5B) and LTD (Fig 5C) were considered. Because the expression of synaptic plasticity closely relies on NMDAR activation, changes in high-frequency stimulation-induced LTP suggest an effect of AR^NesCre^ mutation on the activation of this subtype of glutamate receptors.

#### NMDA receptor activation

**Fig 6:**
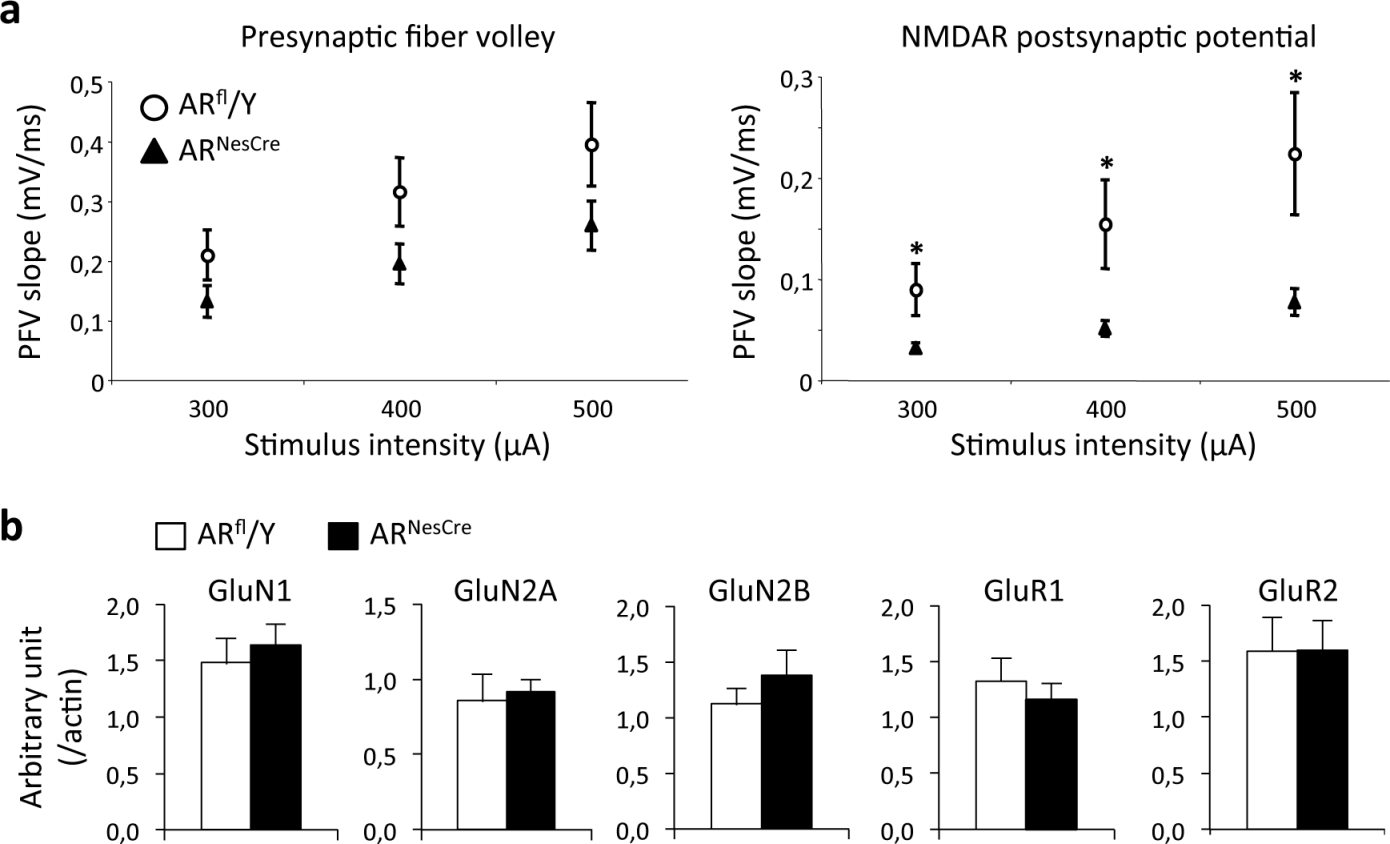
NMDAR-mediated synaptic activation in the hippocampal CA1 area of AR^fl^/Y and AR^NesCre^ male mice. (**a**) Input/Output (I/O) curves of presynaptic fiber volleys (**left**) and NMDAR-mediated fEPSPs (**right**) in low-magnesium medium supplemented with 2,3-dioxo-6-nitro-1,2,3,4-tetrahydrobenzoquinoxaline-7-sulfonamide (12–13 slices from 8 males per genotype). PFV magnitude was not affected, whereas fEPSP values were significantly lower at all stimulus intensities in AR^NesCre^ males (*p < 0.05). (**b)** Densitometric quantification of NMDAR (GluN1, GluN2A, GluN2B) and non-NMDAR (GluR1, GluR2) proteins normalized to ß-actin in the CA1 area (n = 6–8 males per genotype).

In low-Mg^2+^ medium in which non-NMDAR were blocked, a comparison of input/output curves showed that presynaptic fiber volleys were not affected in AR^NesCre^ males whatever the stimulus intensity, whereas NMDAR-mediated field excitatory postsynaptic potentials were significantly reduced (Fig 6A). Thus, the neural deletion of *AR* impacts NMDAR activation through changes affecting postsynaptic receptors located on pyramidal cells.

### Quantification of glutamate receptors in the CA1 region

Analysis of the proteins extracted from the CA1 area showed that the levels of NMDA (GluN1, GluN2A and GluN2B) and α-amino-3-hydroxy-5-methyl-4-isoxazolepropionic acid (AMPA) receptor subunits (GluR1 and GluR2) were similar (*p* > 0.05) in AR^NesCre^ and AR^fl^/Y males (Fig 6B). These results indicate that the altered NMDAR activation was not due to changes in receptor protein levels in the CA1 area.

## Discussion

We used the mouse line lacking the *AR* specifically in the nervous system [33] in order to analyze the effects of neural *AR* mutation on temporal order memory for visual objects and novel object recognition. Using this mouse line presents advantages over pharmacological studies and the *Tfm* model. Neural *AR* mutants, unlike *Tfm* animals, display normal development of the urogenital tract and have functional testes [33]. By contrast to gonadectomy and dihydrotestosterone supplementation, in the AR^NesCre^ model both gonad androgens and neurosteroids are unable to activate the neural AR since *AR* gene is invalidated in the whole nervous system including the hippocampus ([33]; present study). Indeed, locally synthesized sex steroids have been reported to modulate hippocampal synaptic plasticity and healthy memory processes [45]. Furthermore, dihydrotestosterone does not exclusively activate the AR, as it can be metabolized to 5α-androstan-3ß, which binds estrogen receptors and triggers neural responses [46].

We found that AR^NesCre^ males were unable to discriminate between the first and most recent object seen, despite a normal detection of visual object novelty. Whether this deficit is due to impaired consolidation for the earlier or last object seen [22,47] needs further investigation. Previous studies reported a decrease in the exploration of new objects and increases in anxiety indices in androgen-insensitive mice carrying the testicular feminization mutation [48]. In our study, mutants displayed unchanged anxiety-related behavior and circadian secretion of glucocorticoids. Unlike *AR* conditional mutants, *Tfm* mutation results in cryptorchidic and azoospermic testes and low levels of circulating testosterone [49]. It is thus possible that decreased gonadal testosterone in *Tfm* mice results in reduced neural aromatization of testosterone into estradiol, thereby interfering also with the estrogenic regulation of these behaviors. In gonadectomized and testosterone-supplemented AR^NesCre^ males, the unchanged locomotor activity [33], anxiety-related behavior and interest in new objects (present study) may be related to a normally functional estrogenic pathway. We have previously reported that the number of ER-immunoreactive neurons was either unaffected in the bed nucleus of stria terminalis and septum, or increased in the medial amygdala and medial preoptic area of AR^NesCre^ males [33,50]. The present data showing similar amounts of hippocampal ERα protein in control and mutant males support the hypothesis that the behavioral deficits induced by the AR^NesCre^ mutation may not be caused by an indirect effect on estrogen receptors.

Given the specific role of the CA1 area in the temporal processing of visual objects and the high level of AR expression in this hippocampal region [32,33], we hypothesized that AR^NesCre^ mutation may affect the processing of temporal information by altering synaptic activities in this hippocampal area. Electrophysiological analysis indicated that AR^NesCre^ mutation impaired basal glutamate synaptic transmission and high-frequency stimulation-induced LTP expression, while sparing theta-burst conditioning stimulation-dependent LTP and LTD induction in the CA1 area. These effects are opposite to those recorded in the same region of young and adolescent rats with a pharmacological blockade of the AR during stimulation protocols [51]. Although these experimental conditions are far to be comparable, it is possible that the deficits characterized in our model result from early effects of the AR^NesCre^ mutation during development and puberty, potentially accounting for this discrepancy. Exposure to gonadal testosterone during development and puberty is known to have long-term effects on the behavioral and physiological functions of the hippocampus [52,53]. Interestingly, AR^NesCre^ mutation triggers *AR* deletion by embryonic day 10.5, before gonadal differentiation and testosterone synthesis have occurred. Alternatively, AR^NesCre^ mutation may also have late effects during adulthood, because the role of the hippocampal AR seems to increase in importance during adulthood [54].

Our results indicate that neural *AR* deletion affected high-frequency stimulation-induced LTP driven by sustained NMDAR activation. This could underlie the defect of the temporal processing of visual objects in mutant males. Consistent with this hypothesis, NMDAR in CA1 pyramidal cells, the GluN1 subunits in particular, have been shown to play a critical role in the formation of temporal memory [17]. We found that the deficits of NMDAR activation occurred principally at the postsynaptic level, since the magnitude of the NMDAR-mediated field excitatory postsynaptic potentials were decreased whereas activation of presynaptic fiber volleys was not affected. The normal levels of protein for NMDAR subunits in the CA1 of AR^NesCre^ males strongly suggest that the impaired activation of NMDAR is rather due to changes in the functional or pharmacological properties of the receptor, as reported for anabolic androgens [55]. It is important to note that neural *AR* deletion had differential effects on the expression of NMDAR-dependent synaptic plasticity depending on the strength of the presynaptic activation. Theta-burst conditioning stimulation-induced LTP or LTD was not affected in mutant mice probably because the low degree of NMDAR activation required for their expression could still be achieved after neural *AR* deletion. Alternately, compensatory mechanisms could occur to alleviate the altered NMDAR activation such as the involvement of calcium channels, as observed for example in physiological aging [56,57]. On the contrary, the mutation could restrain the degree of sustained NMDAR activation induced by high-frequency stimulation or compensatory mechanisms could be saturated in this case, thus reducing the magnitude of the related LTP. Our results therefore suggest that testosterone may act through the AR to regulate the degree of synaptic plasticity within CA1 networks.

The data presented here strongly suggest that the impaired temporal processing of information in mutant mice may be due to changes in glutamate transmission in the hippocampal CA1 area, although possible changes in the cortex cannot be ruled out [20]. The loss or down-regulation of neural AR may be detrimental to functions and behaviors implemented by these brain areas, thus constituting a risk factor. In humans, testosterone levels have been linked to performance in visual and episodic memory tasks, with hypogonadic and elderly men performing poorly in such tasks [8,9,58]. Age-related deficits have also been reported for temporal processes in rats [59]. However, variable effects have been reported for androgen replacement therapy in men [60]. A recent study showed that dihydrotestosterone supplementation in rats attenuated the increase in functional plasticity triggered by gonadectomy, for mossy fibers in the CA3 area [37]. This suggests that the androgen effects mediated by the AR may display regional specificity. This specificity, together with the extent of hippocampal damage, may underlie the variability of the effects reported for androgen replacement therapy in men.

In conclusion, we provide the first direct evidence that the neural AR is critical for the processing of temporal information for visual objects, possibly through the modulation of glutamatergic transmission in the hippocampal CA1 area. Our genetic model could be used to decipher neural AR mechanisms and open up new perspectives in the design of brain-selective AR modulators for use in the prevention or treatment of diseases associated with an impairment of temporal memory. A recent transcriptome meta-analysis revealing the key role of AR in Alzheimer’s disease [61] highlights the relevance of such an approach.

## References

[1] Janowsky JS. Thinking with your gonads: testosterone and cognition. Trends Cogn Sci. 2006; 10: 77–82. 10.1016/j.tics.2005.12.010 16386941

[2] Galea LAM, Uban KA, Epp JR, Brummelte S, Barha CK, Wilson WL, et al Endocrine regulation of cognition and neuroplasticity: our pursuit to unveil the complex interaction between hormones, the brain, and behaviour. Can J Exp Psychol Rev Can Psychol Expérimentale. 2008; 62: 247–260. 10.1037/a0014501 19071993

[3] Howell S, Shalet S. Testosterone deficiency and replacement. Horm Res. 2001; 56 Suppl 1: 86–92. 11786693 10.1159/000048142

[4] Cherrier MM, Anawalt BD, Herbst KL, Amory JK, Craft S, Matsumoto AM, et al Cognitive effects of short-term manipulation of serum sex steroids in healthy young men. J Clin Endocrinol Metab. 2002; 87: 3090–3096. 10.1210/jcem.87.7.8570 12107206

[5] Lamberts SW, van den Beld AW, van der Lely AJ. The endocrinology of aging. Science. 1997; 278: 419–424. 9334293 10.1126/science.278.5337.419

[6] Harman SM, Metter EJ, Tobin JD, Pearson J, Blackman MR, Baltimore Longitudinal Study of Aging. Longitudinal effects of aging on serum total and free testosterone levels in healthy men. Baltimore Longitudinal Study of Aging. J Clin Endocrinol Metab. 2001; 86: 724–731. 10.1210/jcem.86.2.7219 11158037

[7] Alexander GM, Swerdloff RS, Wang C, Davidson T, McDonald V, Steiner B, et al Androgen–Behavior Correlations in Hypogonadal Men and Eugonadal Men. Horm Behav. 1998; 33: 85–94. 10.1006/hbeh.1998.1439 9647934

[8] Moffat SD, Zonderman AB, Metter EJ, Blackman MR, Harman SM, Resnick SM. Longitudinal assessment of serum free testosterone concentration predicts memory performance and cognitive status in elderly men. J Clin Endocrinol Metab. 2002; 87: 5001–5007. 10.1210/jc.2002-020419 12414864

[9] Thilers PP, Macdonald SWS, Herlitz A. The association between endogenous free testosterone and cognitive performance: a population-based study in 35 to 90 year-old men and women. Psychoneuroendocrinology. 2006; 31: 565–576. 10.1016/j.psyneuen.2005.12.005 16487665

[10] Ceccarelli I, Scaramuzzino A, Aloisi AM. Effects of gonadal hormones and persistent pain on non-spatial working memory in male and female rats. Behav Brain Res. 2001; 123: 65–76. 11377730 10.1016/s0166-4328(01)00195-4

[11] Frye CA, Edinger KL, Seliga AM, Wawrzycki JM. 5alpha-reduced androgens may have actions in the hippocampus to enhance cognitive performance of male rats. Psychoneuroendocrinology. 2004; 29: 1019–1027. 10.1016/j.psyneuen.2003.10.004 15219653

[12] Edinger KL, Frye CA. Testosterone’s analgesic, anxiolytic, and cognitive-enhancing effects may be due in part to actions of its 5alpha-reduced metabolites in the hippocampus. Behav Neurosci. 2004; 118: 1352–1364. 10.1037/0735-7044.118.6.1352 15598144

[13] Gibbs RB, Johnson DA. Sex-specific effects of gonadectomy and hormone treatment on acquisition of a 12-arm radial maze task by Sprague Dawley rats. Endocrinology. 2008; 149: 3176–3183. 10.1210/en.2007-1645 18292188 PMC2408814

[14] Pause BM, Zlomuzica A, Kinugawa K, Mariani J, Pietrowsky R, Dere E. Perspectives on episodic-like and episodic memory. Front Behav Neurosci. 2013; 7: 33 10.3389/fnbeh.2013.00033 23616754 PMC3629296

[15] Hopkins RO, Kesner RP, Goldstein M. Item and order recognition memory in subjects with hypoxic brain injury. Brain Cogn. 1995; 27: 180–201. 10.1006/brcg.1995.1016 7772332

[16] Downes JJ, Mayes AR, MacDonald C, Hunkin NM. Temporal order memory in patients with Korsakoff’s syndrome and medial temporal amnesia. Neuropsychologia. 2002; 40: 853–861. 11900736 10.1016/s0028-3932(01)00172-5

[17] Huerta PT, Sun LD, Wilson MA, Tonegawa S. Formation of temporal memory requires NMDA receptors within CA1 pyramidal neurons. Neuron. 2000; 25: 473–480. 10719900 10.1016/s0896-6273(00)80909-5

[18] Hannesson DK, Howland JG, Phillips AG. Interaction between perirhinal and medial prefrontal cortex is required for temporal order but not recognition memory for objects in rats. J Neurosci. 2004; 24: 4596–4604. 10.1523/JNEUROSCI.5517-03.2004 15140931 PMC6729404

[19] Kesner RP, Hunsaker MR, Ziegler W. The role of the dorsal CA1 and ventral CA1 in memory for the temporal order of a sequence of odors. Neurobiol Learn Mem. 2010; 93: 111–116. 10.1016/j.nlm.2009.08.010 19733676

[20] Barker GRI, Warburton EC. When is the hippocampus involved in recognition memory? J Neurosci. 2011; 31: 10721–10731. 10.1523/JNEUROSCI.6413-10.2011 21775615 PMC6622630

[21] Kesner RP, Hunsaker MR. The temporal attributes of episodic memory. Behav Brain Res. 2010; 215: 299–309. 10.1016/j.bbr.2009.12.029 20036694

[22] Hoge J, Kesner RP. Role of CA3 and CA1 subregions of the dorsal hippocampus on temporal processing of objects. Neurobiol Learn Mem. 2007; 88: 225–231. 10.1016/j.nlm.2007.04.013 17560815 PMC2095779

[23] Hunsaker MR, Kesner RP. Evaluating the differential roles of the dorsal dentate gyrus, dorsal CA3, and dorsal CA1 during a temporal ordering for spatial locations task. Hippocampus. 2008; 18: 955–964. 10.1002/hipo.20455 18493930 PMC2570230

[24] Packard MG, Kohlmaier JR, Alexander GM. Posttraining intrahippocampal estradiol injections enhance spatial memory in male rats: interaction with cholinergic systems. Behav Neurosci. 1996; 110: 626–632. 8889009 10.1037//0735-7044.110.3.626

[25] Kramár EA, Chen LY, Brandon NJ, Rex CS, Liu F, Gall CM, et al Cytoskeletal changes underlie estrogen’s acute effects on synaptic transmission and plasticity. J Neurosci. 2009; 29: 12982–12993. 10.1523/JNEUROSCI.3059-09.2009 19828812 PMC2806054

[26] González-Burgos I, Rivera-Cervantes MC, Velázquez-Zamora DA, Feria-Velasco A, Garcia-Segura LM. Selective estrogen receptor modulators regulate dendritic spine plasticity in the hippocampus of male rats. Neural Plast. 2012; 2012: 309494 10.1155/2012/309494 22164341 PMC3216374

[27] Tsurugizawa T, Mukai H, Tanabe N, Murakami G, Hojo Y, Kominami S, et al Estrogen induces rapid decrease in dendritic thorns of CA3 pyramidal neurons in adult male rat hippocampus. Biochem Biophys Res Commun. 2005; 337: 1345–1352. 10.1016/j.bbrc.2005.09.188 16242668

[28] Tanaka M, Sokabe M. Bidirectional modulatory effect of 17ß-estradiol on NMDA receptors via ERα and ERß in the dentate gyrus of juvenile male rats. Neuropharmacology. 2013; 75: 262–273. 10.1016/j.neuropharm.2013.07.029 23954493

[29] Liu F, Day M, Muñiz LC, Bitran D, Arias R, Revilla-Sanchez R, et al Activation of estrogen receptor-beta regulates hippocampal synaptic plasticity and improves memory. Nat Neurosci. 2008; 11: 334–343. 10.1038/nn2057 18297067

[30] Day M, Sung A, Logue S, Bowlby M, Arias R. Beta estrogen receptor knockout (BERKO) mice present attenuated hippocampal CA1 long-term potentiation and related memory deficits in contextual fear conditioning. Behav Brain Res. 2005; 164: 128–131. 10.1016/j.bbr.2005.05.011 16054246

[31] Cho J, Yu N-K, Choi J-H, Sim S-E, Kang SJ, Kwak C, et al Multiple repressive mechanisms in the hippocampus during memory formation. Science. 2015; 350: 82–87. 10.1126/science.aac7368 26430118

[32] Kerr JE, Allore RJ, Beck SG, Handa RJ. Distribution and hormonal regulation of androgen receptor (AR) and AR messenger ribonucleic acid in the rat hippocampus. Endocrinology. 1995; 136: 3213–3221. 10.1210/endo.136.8.7628354 7628354

[33] Raskin K, de Gendt K, Duittoz A, Liere P, Verhoeven G, Tronche F, et al Conditional Inactivation of Androgen Receptor Gene in the Nervous System: Effects on Male Behavioral and Neuroendocrine Responses. J Neurosci. 2009; 29: 4461–4470. 10.1523/JNEUROSCI.0296-09.2009 19357272 PMC6665718

[34] Leranth C, Petnehazy O, MacLusky NJ. Gonadal hormones affect spine synaptic density in the CA1 hippocampal subfield of male rats. J Neurosci. 2003; 23: 1588–1592. 12629162 10.1523/JNEUROSCI.23-05-01588.2003 PMC6741990

[35] Hajszan T, MacLusky NJ, Johansen JA, Jordan CL, Leranth C. Effects of androgens and estradiol on spine synapse formation in the prefrontal cortex of normal and testicular feminization mutant male rats. Endocrinology. 2007; 148: 1963–1967. 10.1210/en.2006-1626 17317772 PMC2128740

[36] Pouliot WA, Handa RJ, Beck SG. Androgen modulates N-methyl-D-aspartate-mediated depolarization in CA1 hippocampal pyramidal cells. Synap N Y N. 1996; 23: 10–19. 10.1002/(SICI)1098-2396(199605)23:1<10::AID-SYN2>3.0.CO;2-K 8723131

[37] Skucas VA, Duffy AM, Harte-Hargrove LC, Magagna-Poveda A, Radman T, Chakraborty G, et al Testosterone depletion in adult male rats increases mossy fiber transmission, LTP, and sprouting in area CA3 of hippocampus. J Neurosci. 2013; 33: 2338–2355. 10.1523/JNEUROSCI.3857-12.2013 23392664 PMC3711621

[38] Hatanaka Y, Mukai H, Mitsuhashi K, Hojo Y, Murakami G, Komatsuzaki Y, et al Androgen rapidly increases dendritic thorns of CA3 neurons in male rat hippocampus. Biochem Biophys Res Commun. 2009; 381: 728–732. 10.1016/j.bbrc.2009.02.130 19254689

[39] Raskin K, Marie-Luce C, Picot M, Bernard V, Mailly P, Hardin-Pouzet H, et al Characterization of the spinal nucleus of the bulbocavernosus neuromuscular system in male mice lacking androgen receptor in the nervous system. Endocrinology. 2012; 153: 3376–3385. 10.1210/en.2012-1001 22585832

[40] Ennaceur A, Delacour J. A new one-trial test for neurobiological studies of memory in rats. 1: Behavioral data. Behav Brain Res. 1988; 31: 47–59. 3228475 10.1016/0166-4328(88)90157-x

[41] Potier B, Poindessous-Jazat F, Dutar P, Billard JM. NMDA receptor activation in the aged rat hippocampus. Exp Gerontol. 2000; 35: 1185–1199. 11113601 10.1016/s0531-5565(00)00122-4

[42] Anderson WW, Collingridge GL. The LTP Program: a data acquisition program for on-line analysis of long-term potentiation and other synaptic events. J Neurosci Methods. 2001; 108: 71–83. 11459620 10.1016/s0165-0270(01)00374-0

[43] Kollen M, Stéphan A, Faivre-Bauman A, Loudes C, Sinet P-M, Alliot J, et al Preserved memory capacities in aged Lou/C/Jall rats. Neurobiol Aging. 2010; 31: 129–142. 10.1016/j.neurobiolaging.2008.03.010 18462838

[44] Mitchell JB, Laiacona J. The medial frontal cortex and temporal memory: tests using spontaneous exploratory behaviour in the rat. Behav Brain Res. 1998; 97: 107–113. 9867236 10.1016/s0166-4328(98)00032-1

[45] Ooishi Y, Kawato S, Hojo Y, Hatanaka Y, Higo S, Murakami G, et al Modulation of synaptic plasticity in the hippocampus by hippocampus-derived estrogen and androgen. J Steroid Biochem Mol Biol. 2012; 131: 37–51. 10.1016/j.jsbmb.2011.10.004 22075082

[46] Handa RJ, Pak TR, Kudwa AE, Lund TD, Hinds L. An alternate pathway for androgen regulation of brain function: activation of estrogen receptor beta by the metabolite of dihydrotestosterone, 5alpha-androstane-3beta,17beta-diol. Horm Behav. 2008; 53: 741–752. 10.1016/j.yhbeh.2007.09.012 18067894 PMC2430080

[47] Ennaceur A. One-trial object recognition in rats and mice: methodological and theoretical issues. Behav Brain Res. 2010; 215: 244–254. 10.1016/j.bbr.2009.12.036 20060020

[48] Zuloaga DG, Morris JA, Jordan CL, Breedlove SM. Mice with the testicular feminization mutation demonstrate a role for androgen receptors in the regulation of anxiety-related behaviors and the hypothalamic-pituitary-adrenal axis. Horm Behav. 2008; 54: 758–766. 10.1016/j.yhbeh.2008.08.004 18775430

[49] Goldstein JL, Wilson JD. Studies on the pathogenesis of the pseudohermaphroditism in the mouse with testicular feminization. J Clin Invest. 1972; 51: 1647–1658. 10.1172/JCI106966 4402348 PMC292312

[50] Picot M, Naulé L, Marie-Luce C, Martini M, Raskin K, Grange-Messent V, et al Vulnerability of the neural circuitry underlying sexual behavior to chronic adult exposure to oral bisphenol a in male mice. Endocrinology. 2014; 155: 502–512. 10.1210/en.2013-1639 24265451

[51] Pettorossi VE, Di Mauro M, Scarduzio M, Panichi R, Tozzi A, Calabresi P, et al Modulatory role of androgenic and estrogenic neurosteroids in determining the direction of synaptic plasticity in the CA1 hippocampal region of male rats. Physiol Rep. 2013; 1: e00185 10.1002/phy2.185 24744863 PMC3970743

[52] Harley CW, Malsbury CW, Squires A, Brown RA. Testosterone decreases CA1 plasticity in vivo in gonadectomized male rats. Hippocampus. 2000; 10: 693–697. 11153715 10.1002/1098-1063(2000)10:6<693::AID-HIPO1007>3.0.CO;2-G

[53] Isgor C, Sengelaub DR. Effects of neonatal gonadal steroids on adult CA3 pyramidal neuron dendritic morphology and spatial memory in rats. J Neurobiol. 2003; 55: 179–190. 10.1002/neu.10200 12672016

[54] Kimoto T, Ishii H, Higo S, Hojo Y, Kawato S. Semicomprehensive analysis of the postnatal age-related changes in the mRNA expression of sex steroidogenic enzymes and sex steroid receptors in the male rat hippocampus. Endocrinology. 2010; 151: 5795–5806. 10.1210/en.2010-0581 21047951

[55] Rossbach UL, Le Grevès M, Nyberg F, Zhou Q, Le Grevès P. Acute 19-nortestosterone transiently suppresses hippocampal MAPK pathway and the phosphorylation of the NMDA receptor. Mol Cell Endocrinol. 2010; 314: 143–149. 10.1016/j.mce.2009.07.027 19660519

[56] Shankar S, Teyler TJ, Robbins N. Aging differentially alters forms of long-term potentiation in rat hippocampal area CA1. J Neurophysiol. 1998; 79: 334–341. 9425202 10.1152/jn.1998.79.1.334

[57] Junjaud G, Rouaud E, Turpin F, Mothet J-P, Billard J-M. Age-related effects of the neuromodulator D-serine on neurotransmission and synaptic potentiation in the CA1 hippocampal area of the rat. J Neurochem. 2006; 98: 1159–1166. 10.1111/j.1471-4159.2006.03944.x 16790028

[58] Golomb JD, Peelle JE, Addis KM, Kahana MJ, Wingfield A. Effects of adult aging on utilization of temporal and semantic associations during free and serial recall. Mem Cognit. 2008; 36: 947–956. 18630201 10.3758/mc.36.5.947 PMC2839458

[59] Hauser E, Tolentino JC, Pirogovsky E, Weston E, Gilbert PE. The effects of aging on memory for sequentially presented objects in rats. Behav Neurosci. 2009; 123: 1339–1345. 10.1037/a0017681 20001117 PMC2819214

[60] Driscoll I, Resnick SM. Testosterone and cognition in normal aging and Alzheimer’s disease: an update. Curr Alzheimer Res. 2007; 4: 33–45. 17316164 10.2174/156720507779939878

[61] Winkler JM, Fox HS. Transcriptome meta-analysis reveals a central role for sex steroids in the degeneration of hippocampal neurons in Alzheimer’s disease. BMC Syst Biol. 2013; 7: 51 10.1186/1752-0509-7-51 23803348 PMC3702487

